# Implementing the organizational readiness for change survey during a novel midwifery preceptor program in Sierra Leone: stakeholder results

**DOI:** 10.1186/s12913-024-11435-9

**Published:** 2024-08-21

**Authors:** Brittney J. van de Water, Ashley H. Longacre, Jenny Hotchkiss, Mustapha Sonnie, Julie Mann, Elizabeth Lemor

**Affiliations:** 1 Seed Global Health, Boston, MA USA; 2 Boston College Connell School of Nursing, 140 Commonwealth Avenue, Chestnut Hill, MA 02467 USA; 3 Boston College, Chestnut Hill, MA USA; 4 Seed Global Health, Freetown, Sierra Leone; 5 Mount Auburn Hospital, Cambridge, MA USA; 6 Ministry of Health Sierra Leone, Freetown, Sierra Leone

**Keywords:** Clinical learning environment, Midwifery readiness for change, Stakeholder engagement, Implementation science

## Abstract

**Background:**

Sierra Leone has one of the world’s highest maternal and infant mortality rates and suffers from a shortage of well-trained health professionals, including midwives. Prior to engaging in systematic interventions, it is critical to measure organizational readiness to gauge members’ psychological and behavioral preparedness to implement change. We aimed to measure the organizational readiness for implementing change and compare results among midwives and administrative leaders at two schools of midwifery in Sierra Leone prior to the rollout of a midwifery preceptor program.

**Methods:**

The Organizational Readiness for Implementing Change (ORIC) survey is a validated 12-item questionnaire designed to assess two domains of organizational readiness for change: *change commitment (motivation)* and *change efficacy (capacity)*. All survey items begin with the same prompt and a five-item Likert scale response, with seven questions about change commitment and five about change efficacy. Data collection occurred in two schools of midwifery in Sierra Leone during two day-long meetings with stakeholders. Statistical analysis was conducted using descriptive statistics and Wilcoxon rank-sum test to compare independent samples: School 1 versus School 2 (site), midwife versus other roles (role).

**Results:**

Participants included 42 respondents (mean age 41 years, 95% female). Surveys were distributed evenly between the two sites. Occupations included midwifery faculty (*n* = 8), administrators (*n* = 5), clinicians (*n* = 25), and clinical educators (*n* = 4). Domain 1 (change commitment) had a mean score of 4.72 (SD 0.47) while Domain 2 (change efficacy) had a mean score of 4.53 (SD 0.54) out of a total potential score of five. There were no statistically significant differences between site responses for Domain 1 (*p* = 0.5479) and Domain 2 (*p* = 0.1026) nor role responses for Domain 1 (*p* = 0.0627) and Domain 2 (*p* = 0.2520).

**Conclusion:**

Stakeholders had very high overall readiness for change across all ORIC questions for both change commitment and change efficacy. Mean scores for change commitment were slightly higher which is not surprising given the low-resourced settings stakeholders work in while training students. High mean scores across sites and roles is encouraging as this novel preceptor program is currently being rolled out.

**Supplementary Information:**

The online version contains supplementary material available at 10.1186/s12913-024-11435-9.

## Background

Maternal and infant mortality in Sierra Leone is exceedingly high with 443 per 100,000 mothers dying and 3,900 per 100,000 live births ending in infant death [1]. Due to these devastating statistics, organizational change is necessary to transform maternal and infant care in the country. Recent studies have reported the need to increase the number of midwives in Sierra Leone, but also to improve competence and confidence of midwives [1, 2].

Additionally, the clinical learning environment for midwifery students in Sierra Leone could be improved. The Sierra Leone Ministry of Health and Seed Global Health, a health non-government organization, formed a partnership in 2020 to improve midwifery education in the country, starting with two schools of Midwifery in separate districts in Sierra Leone. Seed conducted a needs assessment in Sierra Leone and found the clinical learning environment often lacks infrastructure and resources, as well as preceptors − 52% of midwifery students who attended a birth during their clinical placement did so without a preceptor present [3]. However, midwives and hospital management report the need for additional midwifery training and being motivated by professional development opportunities [3]. When assessing clinical learning environments for midwifery students, the placement site, specifically a tertiary hospital versus clinic, impacts students’ learning experience [4, 5]. In Sierra Leone, clinics were found to offer students significantly more supportive environments to practice and develop skills and agreed preceptors treated them with significantly more respect, helped improve their skills, provided a safe environment to ask questions, and had stronger teaching and mentorship skills compared to students placed at hospitals [4]. Therefore, understanding the organizational readiness to implement change among prospective stakeholders (midwifery preceptors and midwifery school and hospital management) is important prior to initiating a novel preceptor program aimed to improve precepting skills (i.e. competence and confidence) for midwives who are to support students in the clinical learning environment.

Organizational readiness is paramount for interventions to work - roughly half of all implementation failures are due to leaders failing to establish organizational readiness for change [6]. Organizational readiness for implementing change is the ‘organizational members’ psychological and behavioral preparedness to implement change and is a critical precursor to successful implementation of complex change in healthcare’ according to Weiner’s theory of organizational readiness for change [7]. Therefore, the aim in this study was to understand stakeholders organizational readiness for implementing change and compare scores across sites and roles during two day-long preceptor program launch meetings prior to implementing a novel midwifery preceptor program.

## Methods

### Study design

ORIC is a 12-item questionnaire designed to assess two dimensions of organizational readiness for change: *change commitment* (motivation) and *change efficacy (*capacity*)*. It is based directly on Weiner’s Theory of organizational readiness for change theory [7]. The survey has good construct validity (with change commitment scale and change efficacy scale alpha coefficients 0.91 and 0.89 respectively); and needs additional predictive validity [6]. All items start with the same prompt and seven questions ask about change commitment and five ask about change efficacy. Additional file 1.

### Organizational readiness for change theory

The theory of organizational readiness for change consists of two dimensions: change commitment and change efficacy [7]. Both dimensions are related to shared belief with change commitment focusing more on motivation to change and change efficacy focusing more on one’s capacity to change [7]. Organizational readiness for change can foster change-related efforts including initiation, persistence, and cooperative behavior - all leading to improved implementation effectiveness [7]. Upstream contextual factors include organizational culture, policies and procedures, past experience, organization resources, and organizational structure can also all influence readiness to implement change [7].

### Change commitment and change efficacy domains

Change commitment can be motivated by three reasons: because organizational members want to (they value the change), they have to (they have little choice), or because they ought to (they feel obligated). Comparing these three motivations for organizational change, ‘want to’ motivation reflects the highest level of commitment [7, 8]. In the ORIC survey, questions 1, 2, 4, 6, 7, 9, and 11 focus on change commitment. Additionally, according to Weiner, change efficacy refers to organizational members’ shared beliefs in collective capabilities to organize and execute a course of action to implement change, similar to Bandura’s notion of collective efficacy [7, 9]. The change efficacy domain also draws on social cognitive theory, and relies on three questions: do we know what it will take to implement this change effectively; do we have the resources to implement this change effectively; and can we implement this change effectively given the situation we currently face [7, 10]? Per Weiner’s theory, change efficacy is higher when an organization shares a sense of confidence that, collectively, a complex organizational change can be implemented. In the ORIC survey, questions 3, 5, 8, 10, and 12 focus on change efficacy. Supplement 1 contains the ORIC survey used in this study.

### Setting and sample

This study took place at two public, government schools of midwifery in separate districts in Sierra Leone. The sample included clinical midwives (i.e., practicing midwives without any administrative or teaching responsibilities), midwifery school faculty, hospital administrators and clinical midwifery educators affiliated with the schools of midwifery (i.e., midwives with administrative, leadership and/or teaching responsibilities); all of whom were trained nurse midwives. Clinical midwives are most likely to be the preceptors in the midwifery preceptor program; however, all stakeholder opinions are critical for program success and program delivery across institutions.

### Data collection and materials

The ORIC survey was given to participants during two day-long initial launch meetings for a midwifery preceptor program in two districts in Sierra Leone. The meetings included interactive group process mapping (published elsewhere) to understand the logistics of student clinical site placement and coordination between Schools of Midwifery, hospitals and clinics, and preceptors, an overview of the planned year-long preceptor program and their potential involvement in the program, and participants were asked to anonymously complete ORIC surveys by pen and paper.

### Data analysis

Descriptive statistics were used to describe participant demographics and overall ORIC scores. To assess internal consistency among domain questions, we used Cronbach’s alpha. Because the ORIC data are ordinal, the non-parametric Wilcoxon rank-sum test was used to compare differences between ORIC scores by domain and site (School 1 versus School 2) and domain and role (clinician versus management). Means and standard deviations were reported to provide a sense of the center and dispersion of ORIC responses, and are typically included in existing literature that assesses readiness to change along with results of non-parametric tests [11, 12]. For analysis, we excluded one participant as she was not affiliated with either institution, for a total of 42 participants included for analysis. Statistical analyses were performed using SAS version 9.4 (Cary, NC). All statistical tests were two-sided.

## Results

There was a total of 42 participants including 25 clinicians (60%), 8 midwifery school faculty (19%), 5 hospital administrators (12%), and 4 (10%) clinical educators (Table 1). Half were affiliated with each of the two schools of midwifery and/or associated hospitals or clinics. The majority (95%) were female and the mean age was 41 years (SD 8.32 years).

**Table 1:** Participant demographics

Characteristic	School 1 Frequency (percent)	School 2 Frequency (percent)
Age (mean, SD)	42.65 (9.40)	39.30 (6.90)
Sex		
Male	1 (4.76)	1 (4.76)
Female	20 (95.24)	20 (95.24)
Occupation		
Midwifery School Faculty	4 (19.05)	4 (19.05)
Hospital and Midwifery Administrators	3 (14.29)	2 (9.52)
Midwifery Clinicians	13 (61.90)	12 (57.14)
Midwifery Clinical Educators	1 (4.76)	3 (14.29)

### Organizational readiness for implementing change survey

The overall mean score for domain 1 (change commitment) among all participants was 4.72 (standard deviation 0.47) (Table 2). Scores ranged from a low mean score of 4.60 (SD 0.83) for question 4 to a high mean score of 4.90 (SD 0.30) for question 6. For domain 2 (change efficacy) the mean score was 4.53 (SD 0.53) among all participants. These mean scores ranged from a low mean score of 4.37 (SD 0.80) for question 8 to a high mean score of 4.73 (0.55) for question 5. Cronbach’s alpha for Domain 1 was 0.79 indicating good internal consistency among questions, while Domain 2 Cronbach’s alpha was 0.66, indicating acceptable consistency among questions. The intraclass correlation coefficient between the two domains was 0.69, indicating moderate reliability [13, 14].

**Table 2:** Frequency (%) and mean (SD) ORIC survey results

	1- Disagree *n*, %	2- Somewhat disagree	3- Neither agree nor disagree	4- Somewhat agree	5- Agree	Mean (SD)
**Domain 1: Change commitment (motivation) (overall domain mean**,** SD)**						**4.72 (0.47**)
Q1 (*n* = 42)	1 (2.38)	0 (0.00)	0 (0.00)	3 (7.14)	38 (90.48)	4.83 (0.66)
Q2 (*n* = 42)	1 (2.38)	1 (2.38)	2 (4.76)	5 (11.90)	33 (78.57)	4.62 (0.88)
Q4 (*n* = 42)	1 (2.38)	1 (2.38)	0 (0.00)	10 (23.81)	30 (71.43)	4.60 (0.83)
Q6 (*n* = 42)	0 (0.00)	0 (0.00)	0 (0.00)	4 (9.52)	38 (90.48)	4.90 (0.30)
Q7 (*n* = 42)	1 (2.38)	0 (0.00)	2 (4.76)	8 (19.05)	31 (73.81)	4.62 (0.79)
Q9 (*n* = 41)	1 (2.44)	0 (0.00)	1 (2.44)	2 (4.88)	37 (90.24)	4.80 (0.71)
Q11 (*n* = 41)	0 (0.00)	0 (0.00)	2 (4.88)	10 (24.39)	29 (70.73)	4.66 (0.57)
**Domain 2: Change efficacy (capacity) (overall domain mean**,** SD)**						**4.53 (0.54)**
Q3 (*n* = 42)	2 (4.76)	2 (4.76)	1 (2.38)	6 (14.29)	31 (73.81)	4.48 (1.09)
Q5 (*n* = 41)	0 (0.00)	0 (0.00)	2 (4.88)	7 (17.07)	32 (78.05)	4.73 (0.55)
Q8 (*n* = 41)	1 (2.44)	0 (0.00)	2 (4.88)	18 (43.90)	20 (48.78)	4.37 (0.80)
Q10 (*n* = 42)	2 (4.76)	0 (0.00)	0 (0.00)	9 (21.43)	31 (73.81)	4.60 (0.91)
Q12 (*n* = 42)	0 (0.00)	0 (0.00)	4 (9.52)	14 (33.33)	24 (57.14)	4.48 (0.67)

### ORIC domain scores by site and role

School 2 site (versus School 2 site) had slightly higher mean scores for both domain 1 with mean score of 4.79 (SD 0.32) versus mean score 4.65 (0.58) (*p* = 0.5462) and domain 2 with mean score 4.68 (SD 0.43) versus mean score 4.39 (0.60) (*p* = 0.0612) (Table 3). Midwifery clinicians and administrators, educators, and other roles had the same mean score for domain 1 of 4.72 (SD 0.56 and 0.31, respectively) (*p* = 0.1015) while midwifery clinicians had slightly higher domain 2 mean scores compared to administrators and educators and faculty roles of 4.57 (SD 0.60) versus mean score 4.48 (SD 0.45) (*p* = 0.2507).

**Table 3:** Wilcoxon rank-sum test by site and role and domain 1 and domain 2 means

	Domain 1 mean (SD)	*p*-value*	Domain 2 mean (SD)	*p*-value*
Site: School 1 (*n* = 21)	4.65 (0.58)	0.5462	4.39 (0.60)	0.0612
Site: School 2 (*n* = 21)	4.79 (0.32)		4.68 (0.43)	
Role: Midwifery clinician (*n* = 25)	4.72 (0.56)	0.1015	4.57 (0.60)	0.2507
Role: Midwifery Administrator / educator / faculty (*n* = 17)	4.72 (0.31)		4.48 (0.45)	

*Obtained from a Wilcoxon rank-sum test

## Discussion

All stakeholders who participated in this organizational readiness for implementing change study had very high mean scores for both domain 1 (change commitment) and domain 2 (change efficacy). Additionally, there were no significant differences in mean scores based on site nor on participant role. Mean score for domain 1 (change commitment) was slightly higher than mean score for domain 2 (change efficacy). This was not surprising given the low resourced settings participants work in to train midwifery students, thus although they report motivation or commitment to implement change, their perceived capacity to change was slightly lower [7, 15]. Additionally, School 2 had slightly higher mean scores for both domains. School 2 is an older institution with a larger faculty, more facilities, and longer standing relationships with the regional hospital compared to School 1, which only opened within the last five years. Per Weiner’s theory, organizational culture can predispose an organization to be better or worse equipped for change. Key values for higher readiness for change include innovation, risk-taking learning, flexibility, and good existing relationships [7]. One reason for slightly higher scores could be the longer existing relationships in School 2 compared to School 1; however, to note, these mean scores were not significantly different. For the best outcome - namely, implementation success - both motivation and confidence for organizational change need to be present, and consistency in the organization is important; intra-organizational variability in readiness perceptions indicate a lower likelihood of implementation success [6, 7]. Variability in perceptions was “acceptable” to “good” in this study [13].

Given such high mean scores across all questions and both domains, the use of the ORIC survey at the outset of implementation for this midwifery preceptor program, when paired with effectiveness evaluations, will help identify if readiness for implementing change is necessary but perhaps not sufficient for effective implementation. This also helped confirm to the authors and stakeholders of this project that participants seem ready to move forward with implementing the project and additional effort is not necessary to prime participants more prior to program implementation.

Two similar studies have taken place in low resource settings regarding midwifery and maternal care services. One study similar to ours was conducted using the ORIC survey among 212 healthcare providers to assess their readiness for change in respectful maternity care practice in Ibadan, Nigeria between 2019 and 2020 and compared with their perceptions on availability of World Health Organization recommended resources for respectful maternal care implementation [16]. Our overall mean scores for domain 1 and 2 were slightly higher than this study found; however, their sample included a much more diverse group of healthcare providers and included nine facilities. Another study, completed in rural South Australia in 2019 assessed readiness for change, using the ORIC survey, among midwives, nurses, and doctors who were transitioning to a new midwifery model of care [17]. This study provided a composite score for all questions rather than a mean score per question or domain; therefore these results are not exactly comparable. The majority of respondents agreed or strongly agreed with all domain 1 questions; however, there was more variability among domain 2 questions with little consistency. This differs from our study where nearly all participants agreed or strongly agreed with each ORIC statement.

Additionally, two studies have assessed precepting and mentorship among nurses using the ORIC survey; however, in different care settings than Sierra Leone. One study assessed preceptors’ perceptions regarding readiness for change among pharmacy students engaging in direct patient care in North Carolina, USA [18]. The authors conducted a mixed-methods study triangulating ORIC results with qualitative interview findings. There was a decline in the perception of change commitment from pre- to post-assessment and emerging themes from the interviews included concerns about varying perceptions regarding contextual factors that could affect implementation as well as the value of early immersion of student pharmacists in health-system practice by preceptor role. This study reveals the importance of sustainment in change commitment and how a pre- and post-design, once an evidence-based practice is implemented, can provide useful insights into stakeholder commitment and concerns to allow for better support engagement and implementation success. Finally, another study assessed mentorship in nurse anesthesia programs to understand how to develop and formalize a mentorship program for nurse anesthesia students in New York, USA [19]. The study evaluated program directors after watching an educational video about stress among student nurse anesthetists and statistically significant increases in median ORIC scores were seen post-educational video. Authors concluded that education was able to increase organization readiness for these constructs of change commitment, change valence, task knowledge, and resource availability. The consideration of educational tools to empower stakeholders with additional knowledge or skills was considered as an option if ORIC scores were lower, and providing knowledge and skills is central to the midwifery preceptor program; however, given our stakeholders high initial ORIC scores, it was unnecessary to provide additional resources or knowledge at the time of the program launch to improve readiness with additional knowledge.

The efforts to scale-up midwifery education in Sierra Leone and globally is not just an issue of graduating more midwives. Upon graduation, midwives must be competent in the International Confederation of Midwives (ICM) Essential Competencies of Midwifery Practice [5]. Access to hands-on, clinical experiences to develop knowledge, skills, and behavior are critical in securing these essential ICM competencies; however, this has repeatedly been identified as a weakness in midwifery education globally [1, 6–10]. Building a strong cohort of midwifery preceptors is critical to ensuring that midwifery students develop the essential ICM competencies. Therefore, assessing organizational readiness across and within organizations where a novel program is to be implemented is useful to understand, and potentially gain, stakeholder motivation and capacity to implement change. Had ORIC scores not been as high across all groups, additional trainings or changes to the devised program could have been created to better fit the needs of the participants. Due to the overall high readiness to implement change among all participants, next steps include choosing preceptors to join the first cohort in each of the respective sites and begin the intensive classroom and clinical skills building portion of the program (full program protocol forthcoming).

### Limitations

As with all studies, this had some limitations. First, there was a lack of heterogeneity between answers and a limited sample size due to convenience sampling and a small program. Additionally, these surveys were completed as part of a program launch, thus we did not include additional questions about midwifery education or qualitative interviews to help triangulate ORIC results at baseline, limiting our ability to examine associations between ORIC results and other perceptions, scales, or information collected from stakeholders involved in this midwifery preceptor program. Similarly, other measures of “readiness” were not included to compare with ORIC results. All of these limitations limited our ability to conduct additional analyses and may limit the generalizability of our findings.

## Conclusion

This is one of the first studies to utilize the ORIC instrument for midwifery precepting practice in sub-Saharan Africa and these results increase the generalizability to similar, low-resourced practice settings. High organizational readiness for implementing change scores were consistent among midwifery stakeholders prior to the launch of a midwifery preceptor program in Sierra Leone. These high mean scores for change commitment and change efficacy indicate the likelihood for early implementation success for this novel midwifery preceptor program.

### Supplementary Information

Supplementary Material 1

## Data Availability

The datasets generated and/or analyzed during the current study are not publicly available but are available from the corresponding author on reasonable request.

## Abbreviations

ICM: International Confederation of Midwifery
ORIC: Organizational Readiness for Implementing Change
SD: Standard deviation
