# Supplementary material for: Implementing the organizational readiness for change survey during a novel midwifery preceptor program in Sierra Leone: stakeholder results
Source: BMC Health Serv Res. 2024 Aug 21;24:961. doi: 10.1186/s12913-024-11435-9 (PMC11337795; doi:10.1186/s12913-024-11435-9)
Supplement: Supplementary file 1 — Supplementary Material 1 [file 12913_2024_11435_MOESM1_ESM.doc]

| Organizational Readiness for Implementing Change (ORIC) Tool Description Organizational Readiness for Implementing Change (ORIC) is a 12-item instrument used to determine how well employees at an organization feel they can implement the change in processes required by a proposed intervention. Each item includes a Likert scale from 1 (Disagree) to 5 (Agree). Specific Instructions This protocol is for *Implementation Science* purposes and should be used when an intervention is planned/being conducted (i.e., pre-implementation phase of a study). It is important to ensure that the survey is administered to multiple individuals within the organization that have different roles relevant to the intervention or decision making around the intervention. This ensures that a broad perspective of opinions are elicited and that barriers/facilitators to the implementation are understood at a variety of levels. |
| --- |

**Pre-survey information**

Organization you work at: ____________________________________________

Your role/job title: __________________________________________________

Age: _____________________

Gender: __________________

**ORIC Survey questions**

1. People who work here feel confident that the organization can get people invested in implementing this change (the Midwifery Preceptor Program).

       1 [ ] Disagree

       2 [ ] Somewhat Disagree

       3 [ ] Neither Agree nor Disagree

       4 [ ] Somewhat Agree

       5 [ ] Agree

2. People who work here are committed to implementing this change (the Midwifery Preceptor Program).

       1 [ ] Disagree

       2 [ ] Somewhat Disagree

       3 [ ] Neither Agree nor Disagree

       4 [ ] Somewhat Agree

       5 [ ] Agree

3. People who work here feel confident that they can keep track of progress in implementing this change (the Midwifery Preceptor Program).

       1 [ ] Disagree

       2 [ ] Somewhat Disagree

       3 [ ] Neither Agree nor Disagree

       4 [ ] Somewhat Agree

       5 [ ] Agree

4. People who work here will do whatever it takes to implement this change (the Midwifery Preceptor Program).

       1 [ ] Disagree

       2 [ ] Somewhat Disagree

       3 [ ] Neither Agree nor Disagree

       4 [ ] Somewhat Agree

       5 [ ] Agree

5. People who work here feel confident that the organization can support people as they adjust to this change (the Midwifery Preceptor Program).

       1 [ ] Disagree

       2 [ ] Somewhat Disagree

       3 [ ] Neither Agree nor Disagree

       4 [ ] Somewhat Agree

       5 [ ] Agree

6. People who work here want to implement this change (the Midwifery Preceptor Program).

       1 [ ] Disagree

       2 [ ] Somewhat Disagree

       3 [ ] Neither Agree nor Disagree

       4 [ ] Somewhat Agree

       5 [ ] Agree

7. People who work here feel confident that they can keep the momentum going in implementing this change (the Midwifery Preceptor Program).

       1 [ ] Disagree

       2 [ ] Somewhat Disagree

       3 [ ] Neither Agree nor Disagree

       4 [ ] Somewhat Agree

       5 [ ] Agree

8. People who work here feel confident that they can handle the challenges that might arise in implementing this change (the Midwifery Preceptor Program).

       1 [ ] Disagree

       2 [ ] Somewhat Disagree

       3 [ ] Neither Agree nor Disagree

       4 [ ] Somewhat Agree

       5 [ ] Agree

9. People who work here are determined to implement this change (the Midwifery Preceptor Program).

       1 [ ] Disagree

       2 [ ] Somewhat Disagree

       3 [ ] Neither Agree nor Disagree

       4 [ ] Somewhat Agree

       5 [ ] Agree

10. People who work here feel confident that they can coordinate tasks so that implementation goes smoothly (the Midwifery Preceptor Program).

       1 [ ] Disagree

       2 [ ] Somewhat Disagree

       3 [ ] Neither Agree nor Disagree

       4 [ ] Somewhat Agree

       5 [ ] Agree

11. People who work here are motivated to implement this change (the Midwifery Preceptor Program).

       1 [ ] Disagree

       2 [ ] Somewhat Disagree

       3 [ ] Neither Agree nor Disagree

       4 [ ] Somewhat Agree

       5 [ ] Agree

12. People who work here feel confident that they can manage the politics of implementing this change (the Midwifery Preceptor Program).

       1 [ ] Disagree

       2 [ ] Somewhat Disagree

       3 [ ] Neither Agree nor Disagree

       4 [ ] Somewhat Agree

       5 [ ] Agree

Protocol source: <https://www.phenxtoolkit.org/protocols/view/310701>

Shea, C. M., Jacobs. S. R., Esserman, D. A., Bruce, K., Y Weiner, B. J. (2014). Organizational readiness for implementing change: a psychometric assessment of a new measure. Implementation Science, 9, 7.
